# Inhomogeneity of noradrenaline levels in syringe pump systems and how to prevent it: an in vitro study

**DOI:** 10.1186/s12871-025-03574-1

**Published:** 2026-01-09

**Authors:** Alexander L. Leibold, Viktoria Kimmerling, Stephanie Vogl, Christoph Dorn, Christoph Eissnert, Richard F. Kraus, Martin G. Kees, Alexander Dejaco

**Affiliations:** 1 Department of Anaesthesiology, University Hospital Regensburg, Franz-Joseph-Strauss-Allee 11, Regensburg, Bavaria 93053 Germany; 2 Hospital Pharmacy, University Hospital Regensburg, Franz-Joseph-Strauss-Allee 11, Regensburg, Bavaria 93053 Germany; 3 Department of Pharmaceutical and Medicinal Chemistry I, University of Regensburg, Universitätsstr. 31, Regensburg, Bavaria 93051 Germany

**Keywords:** Drug mixing methods, Drug dilution, Solution homogeneity, Syringe pump infusions, Medication safety

## Abstract

**Background:**

Accurate drug delivery in intensive care depends on precise and reliable preparation of intravenous solutions. Manual preparation is prone to error, and fluctuations in the delivery of vasoactive agents such as noradrenaline can cause haemodynamic volatility, leading to direct patient harm or being misinterpreted as instability and prompting unnecessary interventions. This study evaluated how different mixing techniques affect the homogeneity of noradrenaline solutions in 50 mL syringes to identify a safe preparation method for clinical use.

**Methods:**

Six preparation methods, combining two target concentrations and three mixing techniques - no mixing; a single end-over-end syringe inversion; and inversion after aspiration of 5 mL air (the “Bubble-Flip”) - were tested in a simulated syringe pump infusion experiment, with a pre-manufactured noradrenaline solution as reference. Each method was tested in five replicates. To illustrate three-dimensional concentration heterogeneity, additional syringes were flash-frozen in liquid nitrogen (four replicates each for two methods). In total, 38 experiments were performed. Noradrenaline concentrations were quantified by high-performance liquid chromatography.

**Results:**

Mixing technique had a marked impact on solution homogeneity. The highest method-level variability (as coefficients of variation) were 30.7 % with no mixing and 12.2 % with a syringe inversion without air. Within-syringe variability ranged overall from 0.61 % to 39.9 %, with the highest values recorded for no mixing (39.0 %, 20.1 %) and inversion without air (14.8 %). By contrast, the Bubble-Flip method and the pre-mixed solution both achieved overall variability ≤2 %, within-syringe variability consistently < 5 %, and no samples deviated more than ±15 % from target. Three-dimensional reconstructions confirmed complex inhomogeneities with pronounced local spikes and drops, highlighting the risks of inadequate mixing.

**Conclusions:**

Commercial pre-mixed products guarantee homogeneity but are costly. Comparable safety and consistency can be achieved with the Bubble-Flip method - a simple syringe inversion in the presence of 5 mL air. In contrast, manual preparation without proper mixing produced unsafe variability. These findings support the adoption of standardised mixing techniques to ensure reliable drug delivery and patient safety, with principles likely generalisable to other intravenously administered drugs requiring dilution.

## Introduction

The accurate preparation and administration of parenteral medications are critical components of patient safety. In high-acuity settings such as anaesthesiology and intensive care, patients routinely receive dozens of medications daily. While precise timing and dosing may be less critical for some drugs, others - such as vasopressors - require to be accurately infused to maintain therapeutic efficacy and meet stable haemodynamic targets. With agents like noradrenaline (NA), even minor deviations in drug concentration or infusion rate can significantly impact a patient’s cardiovascular response. To deliver precise continuous dosing at defined flow rates, syringe pump systems are usually used. However, precise drug delivery depends not only on accurate flow rates but also on the correct, homogeneous, drug concentrations within the syringes. Despite the extensive pharmacological research available on these drugs, there is limited evidence addressing the impact of manual preparation on the homogeneity of drug concentration within syringes, with a potential for harm. Although ready-to-use, pre-filled syringes exist on the market for some medications, they remain limited in availability, and are generally more costly. Consequently, some departments rely on hospital pharmacies to prepare quality-controlled, pre-mixed syringes. In many others, syringes are prepared on-site by manually drawing up drugs from high-concentration vials and diluting them with carrier solutions to achieve the desired concentrations. This manual preparation process is prone to error. Contributing factors include fatigue, stress due to high work load, lack of time and emergency situations, limited operator experience, and mistakes in drug-volume calculations and unit conversions (e.g. mole to gram), as well as faulty preparation techniques [1–6]. Significant discrepancies between labelled and measured drug concentrations have been reported with various manual solution preparation techniques in anaesthetic practice [5, 6]. Studies on mitigation strategies demonstrated that repeated inversion or stirring is an essential step for achieving homogeneous mixtures [7]. Some studies showed considerable inter-individual variability, suggesting that standardized protocols alone may not guarantee consistent results across providers. Rios et al. found inversion of syringes to be superior to shaking in terms of homogeneity, with more than three inversion manoeuvres providing no additional benefit [8].

Garrigue and colleagues demonstrated the benefit of five 180° syringe “shaking motions” (inversions) with presence of an air bubble, compared to no mixing, in achieving homogeneity in 50 mL syringes [9]. They assessed two-dimensional inhomogeneity by taking repeated samples during continuous flow using syringe pumps. With respect to mixing techniques involving an air bubble, they examined only a single approach: solvent first, then drug, then an air bubble, followed by five 180° syringe inversions. Also, their sampling resolution was limited, with measurements taken only every 10 mL, potentially missing local inhomogeneities.

Drug preparation practices differ between institutions, but one common routine is to draw the solvent first and add the drug second. Volume ratios between drug and solvent can significantly vary, depending on the product in use and the target concentration. This sequence, however, is particularly prone to inhomogeneity: introducing a small drug volume into a larger solvent pool generates little turbulence, and mixing efficiency decreases as the drug-to-solvent ratio becomes smaller. In contrast, adding the solvent after the drug inherently promotes better mixing [9]. We hypothesize that even under unfavourable conditions - when a small drug volume is added to a relatively large volume solvent - clinically sufficient homogeneity can be achieved by a single syringe inversion if an air bubble is included. This would provide a fast, practical, and safe method to reduce variability in everyday preparation routines.

The following key questions regarding optimal and safe mixing techniques for time- and dose-critical drugs still remain: How high is the variability of a NA within 50 mL syringes based on common preparation techniques? What is the impact of different mixing procedures on homogeneity? Does the presence of an air bubble with syringe inversion affect mixing quality compared to inverting the syringe without the air bubble? Is a single syringe inversion in the presence of an air bubble sufficient? Are ready-to-use stock solutions of vasopressors more homogeneous than manual preparations?

## Methods

Aim of this study was to determine a safe and practical technique for the preparation of noradrenaline solutions in 50 mL syringes. Through in vitro experiments, we examined the effects of various preparation methods - including different mixing manoeuvres and the presence of an air bubble facilitating mixing - on intra-syringe concentration variability, and compared manual preparations against a pre-mixed commercial formulation. Two types of experiments were carried out: (i) simulation of continuous infusion of NA using syringe pumps to quantify the (in)homogeneity of drug as delivered to the patient, and (ii) flash-freezing of syringes in liquid nitrogen to identify and illustrate the three-dimensional (in)homogeneity (see Table 1).

**Table 1:** Experimental setup of in vitro preparations of noradrenaline solutions

Condition	Preparation method	Mixing technique	Target NA [mg/mL]	NS [mL]	NA 1 mg/mL [mL]	NA 0.1 mg/mL [mL]	Replicates [-]
Pump	M1	No-Mix	0.1	45	5	-	5
	M2	Flip-Only	0.1	45	5	-	5
	M3	Bubble-Flip	0.1	45	5	-	5
	M4	No-Mix	0.01	49.5	0.5	-	5
	M5	Bubble-Flip	0.01	49.5	0.5	-	5
	M6	Pre-Mix	0.1	-	-	50	5
Freeze	M1	No-Mix	0.1	45	5	-	4
	M3	Bubble-Flip	0.1	45	5	-	4

Preparation methods of noradrenaline in 50 mL syringes (M1-M6). No-Mix: no mixing (M1, M4); Flip-Only: forward-back 180° end-over-end syringe inversion (M2); Bubble-Flip: same inversion in the presence of a 5 mL air bubble (M3, M5); Pre-Mix: commercial pre-mixed product (M6). NA: noradrenaline; NS: 0.9 % normal saline solution. Pump: simulation of continuous infusion by syringe pump; Freeze: flash-freezing and three-dimensional characterization

As neither human subjects nor animal subjects were involved and no personal data was processed, formal approval was waived by the institutional ethics board (25-4342-180).

### Materials

- Sodium chloride 0.9 % NS, 500 mL (Braun GmbH, Melsungen, Germany)
- Sinora^®^ 0.1 mg/mL NA, 50 mL (Sintetica GmbH, Mendrisio, Switzerland)
- Sinora^®^ 1 mg/mL NA, 5 mL (Sintetica GmbH, Mendrisio, Switzerland)
- Aspiration Spike, Extra-Spike (Fresenius Kabi, Bad Homburg, Germany)
- Perfusor^®^ Syringe 50 mL with 1.7 × 20 mm aspiration needle (Braun GmbH, Kronberg, Germany)
- Perfusor^®^ Injectomat Line, 150 cm, volume 2.7 mL (Fresenius Kabi, Bad Homburg, Germany)
- Perfusor^®^ Space infusion pump (Braun GmbH, Kronberg, Germany)
- Inject-F Tuberculin syringe, 1 mL (Braun GmbH, Melsungen, Germany)
- Sterican^®^ aspiration/injection needle, 1.2 × 40 mm (Braun GmbH, Kronberg, Germany)

### Solution preparation methods

**Fig. 1:**
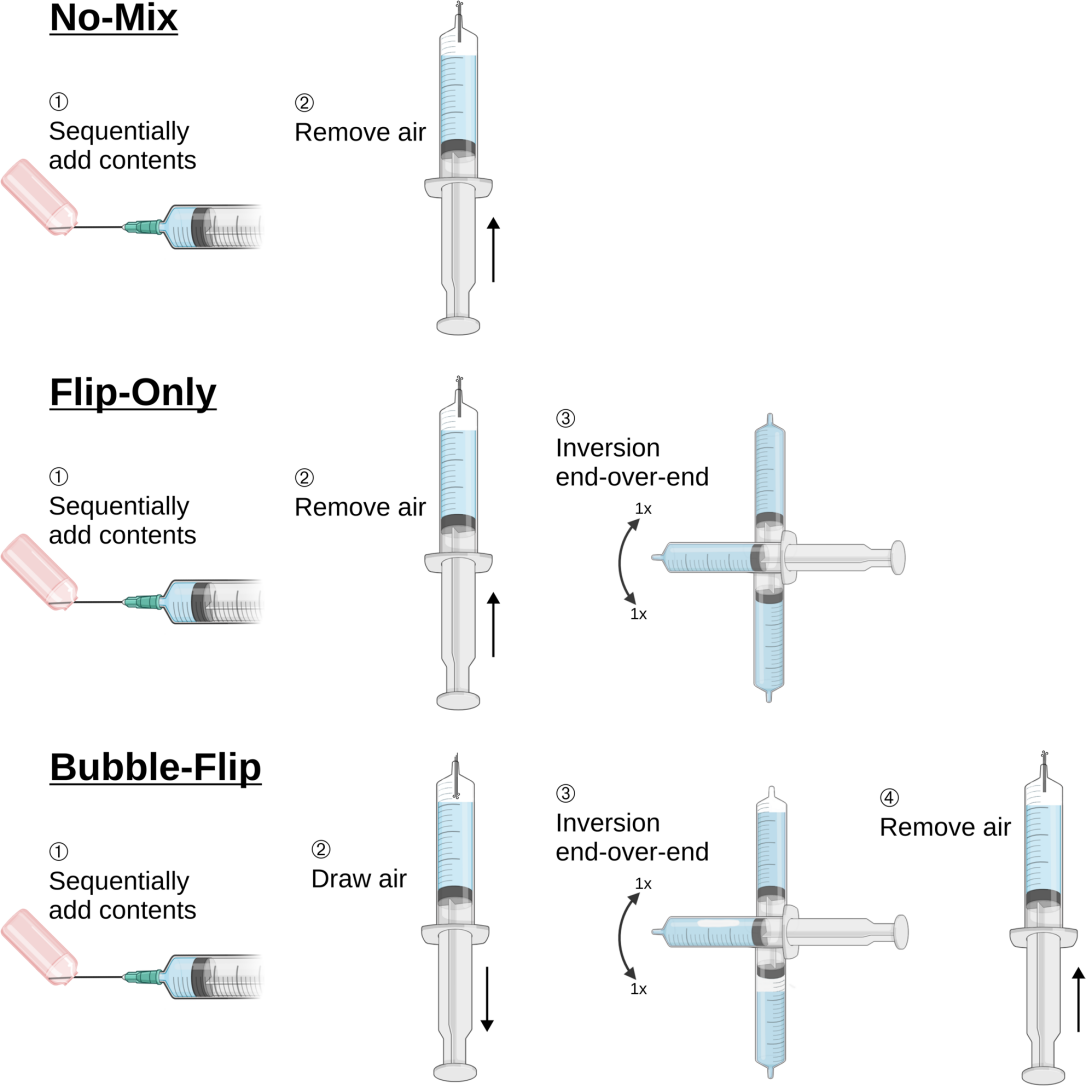
Mixing techniques used to prepare noradrenaline solutions in 50 mL syringes. No-Mix: sequentially add contents, then expel air. Flip-Only: sequentially add contents, remove air, then perform one full 360° end-over-end inversion cycle (180° over, 180° back). Bubble-Flip: sequentially add contents and 5 mL air, perform one full inversion cycle, then remove air. In preparation methods M1-M5, normal saline is drawn first and the drug is added thereafter; only in M6, a pre-mixed noradrenaline solution is used and aspirated directly (see Section “Solution preparation methods”). Illustrations were created using BioRender.com

Six preparation methods (M1-M6) were investigated (Table 1). NA solutions were prepared at two target concentrations (0.1 mg/mL and 0.01 mg/mL). For M1-M5, NA was combined with 0.9 % sodium chloride (normal saline; NS) as solvent. Syringes were held vertically for air aspiration/removal and for NA injection (M4-M5), and horizontally during NA aspiration (M1-M3). The three mixing techniques are illustrated in Fig. 1. M6 consisted of a pre-mixed commercial solution. Key procedural differences between M1–M6, such as the exact sequence in which NS and drug were aspirated, are described below:

- M1 (No-Mix): Aspiration of NS, followed by aspiration of NA, then air removal.
- M2 (Flip-Only): Aspiration of NS, followed by aspiration of NA, air removal, and one full 360° end-over-end inversion cycle (180° over, 180° back).
- M3 (Bubble-Flip): Aspiration of NS, followed by aspiration of NA, aspiration of an air bubble ($\:\approx\:$5 mL), one inversion cycle, and air removal.
- M4 (No-Mix): Aspiration of NS, followed by injection of NA, then air removal.
- M5 (Bubble-Flip): Aspiration of NS, followed by injection of NA, aspiration of an air bubble ($\:\approx\:$5 mL), one inversion cycle, and air removal.
- M6 (Pre-Mix): Aspiration of pre-mixed NA solution from a manufacturer-prepared vial.

### Syringe pump experiments: simulation of continuous infusion

Preparation methods M1-M6 were replicated five times each (S1-S5), leading to *n* = 30 total continuous infusion experiments. All preparations were conducted by three anaesthesiologists according to the same standardised protocol. After preparation, syringes were immediately and carefully inserted into a syringe pump without further agitation. Syringe lines were attached, and pump flow rates were set to 12 mL/h. A timer was started once fluid reached the far end of the syringe lines. Samples of 0.1 mL were taken at the following volumes (1 being the first, and 50 being the last mL of solution to emerge): 1, 2, 3.5, 5, 7, 10, 17, 25.5, 34, 41, 44, 46, 47.5, 49, and 50 mL. To obtain the samples, expelled fluid from the syringe lines was collected over a 30 s period starting at the respective time points (0, 5, 10, 20, 33.3, 50, 96.7, 160, 204.2, 275, 325, 355, 370, 382.5, and 392.5 min). The non-uniform sampling scheme was chosen based on previous reports and our own preliminary experiments which indicated a tendency for concentration variability to be greater at the proximal and distal ends of syringes [9]. Accordingly, sampling density was increased toward both ends to provide higher-resolution data in these regions, while sampling frequency was reduced in the mid-section to account for limited analytical capacity. Samples were stored at -20 °C for less than one month prior to analysis.

### Flash-freeze experiments: three-dimensional assessment

**Fig. 2:**
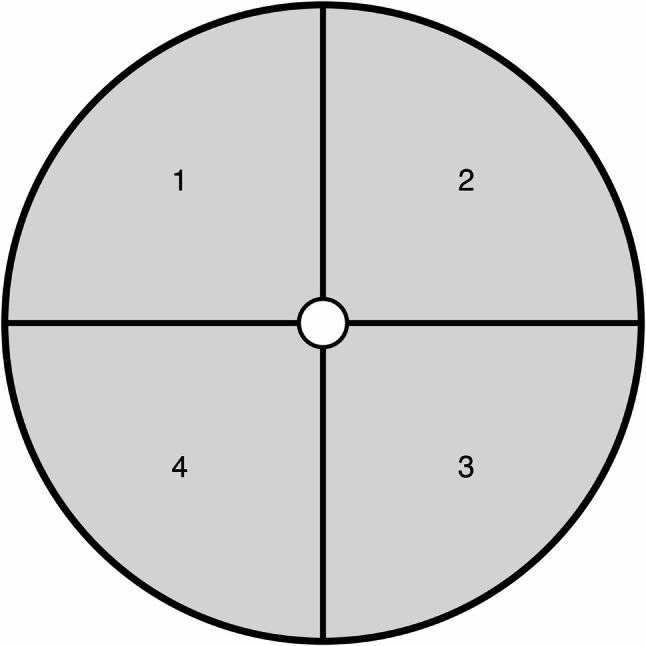
Definition of quadrants 1–4 within frozen syringes, graduation marks facing upwards

Methods M1 and M3 were chosen for rapid cryofixation (flash-freezing) because the preceding syringe-pump experiments indicated that they reliably produced both “good” and “bad” outflow homogeneity, being good candidates allowing us to illustrate both homogeneous and inhomogeneous distributions in three dimensions. Both methods were replicated four times (S1-S4). After preparation, syringes S1-S4 were stored in horizontal position at room temperature for 0, 30, 60, and 120 min, respectively to assess whether a delay between drug solution preparation and freezing affects homogeneity. Syringes were then carefully immersed into liquid nitrogen (-196 °C) with minimal disturbance, still in horizontal position. Freezing was considered complete when no further bubbling was observed, indicating full solidification of the syringe contents. The frozen syringes were then cut transversely into five cylindrical 10 mL segments. The syringe cross-sections’ frozen contents were further divided into four quadrants (see Fig. 2). After thawing and mixing of the respective quadrants from the respective cross-section cylinders, one 0.1 mL sample per quadrant was collected, and NA concentrations quantified high-performance liquid chromatography (HPLC) immediately. A representative axial position within the syringe was assigned based on its segment and quadrant. Specifically, all four measurements per segment were assigned the same axial midpoint (i.e. 5, 15, 25, 35, and 45 mL).

### High-performance liquid chromatography

Quantification of NA concentrations was performed by HPLC using a Shimadzu Nexera-i LC-2040 C system and LabSolution software (Shimadzu Europe, Duisburg, Germany). Detection wavelength was 280 nm. Following injection of 10 µL, separation was performed using a Nucleoshell C18 2.7 μm column (i.d. 100 × 3 mm; Macherey-Nagel, Düren, Germany) at 40 °C and a mobile phase consisting of 0.1 M sodium phosphate buffer (95 %) with 5 mM 1-octansulfonic acid sodium in acetonitrile (5 %) at finally pH 3. The retention time was 3.7 min, and the flow rate was 0.6 mL/min. Intra- and inter-assay inaccuracy and imprecision (five replicates each) at a level of 32 µg/mL were ≤ 2.01 % and ≤ 2.85 %, respectively. The linear calibration was accomplished in the range between 3.9 µg/mL and 1000 µg/mL with an estimated lower limit of quantification of 0.25 µg/mL.

### Statistics and visualisation

Data were processed in LibreOffice Calc 7.3 (The Document Foundation, 2020, Berlin, Germany). Statistics and visualisations were done using R Studio^®^ IDE 12.0 (Posit Software, PBC, Boston, MA, USA) and R project 4.5.1 (R Foundation for Statistical Computing, Vienna, Austria). Three-dimensional plots models were created using the R library rgl. Variability of concentrations ($\:C$) was assessed by coefficient of variation (CV). The CV was defined as $\:CV\mathrm{=}\frac{\sqrt{1/\left(N-1\right){\sum\:}_{1}^{N}{\left(C-\overline{C}\right)}^{2}}}{\overline{C}}\times\:100$, where $\:N$ is the number of samples within a syringe and $\:\overline{C}$ the mean concentration.

In *syringe pump experiments*, to account for non-linear sampling, total drug content in 50 mL syringes was estimated as the area under the concentration-volume curve, and $\:\overline{C}$ was calculated as total drug content divided by syringe volume. To assess variability at the level of preparation methods (M1-M6), repeat experiments (S1-S5) were averaged at each sampling point to obtain a single concentration profile for each method, from which a single overall CV was derived to permit direct comparison across methods. In contrast, within-syringe variability was assessed by calculating the CV directly from the raw concentration values of each syringe without prior averaging. For each preparation method, the syringe with the highest CV was identified as the “least homogeneous” replicate, and its measurements was visualised by boxplots to illustrate worst-case variability. In accordance with the European Pharmacopoeia, individual syringe concentrations were further evaluated against the maximum tolerable deviation of $\:\pm\:$15 % from the target concentration [10]. Clinically relevant deviations were defined as measurements exceeding this threshold, and results are reported as their proportion of all measurements.

In *flash-freeze experiments*, CVs were calculated from all available measurements per syringe (four quadrants per segment, five segments per syringe, 20 samples in total). Because sampling was equidistant, the average concentration $\:\overline{C}$ was obtained as the arithmetic mean of the raw concentrations, and CVs were derived directly using the formula described above. For three-dimensional reconstructions, a continuous gradient was interpolated along the longitudinal axis of the syringe between concentration measurements. Interpolation was restricted to the longitudinal direction since the reconstructions were intended for illustration purposes only and to reduce computational demand. Associations between storage time and within-syringe CV were assessed using ordinary least-squares linear regression.

## Results

### Syringe pump experiments

**Fig. 3:**
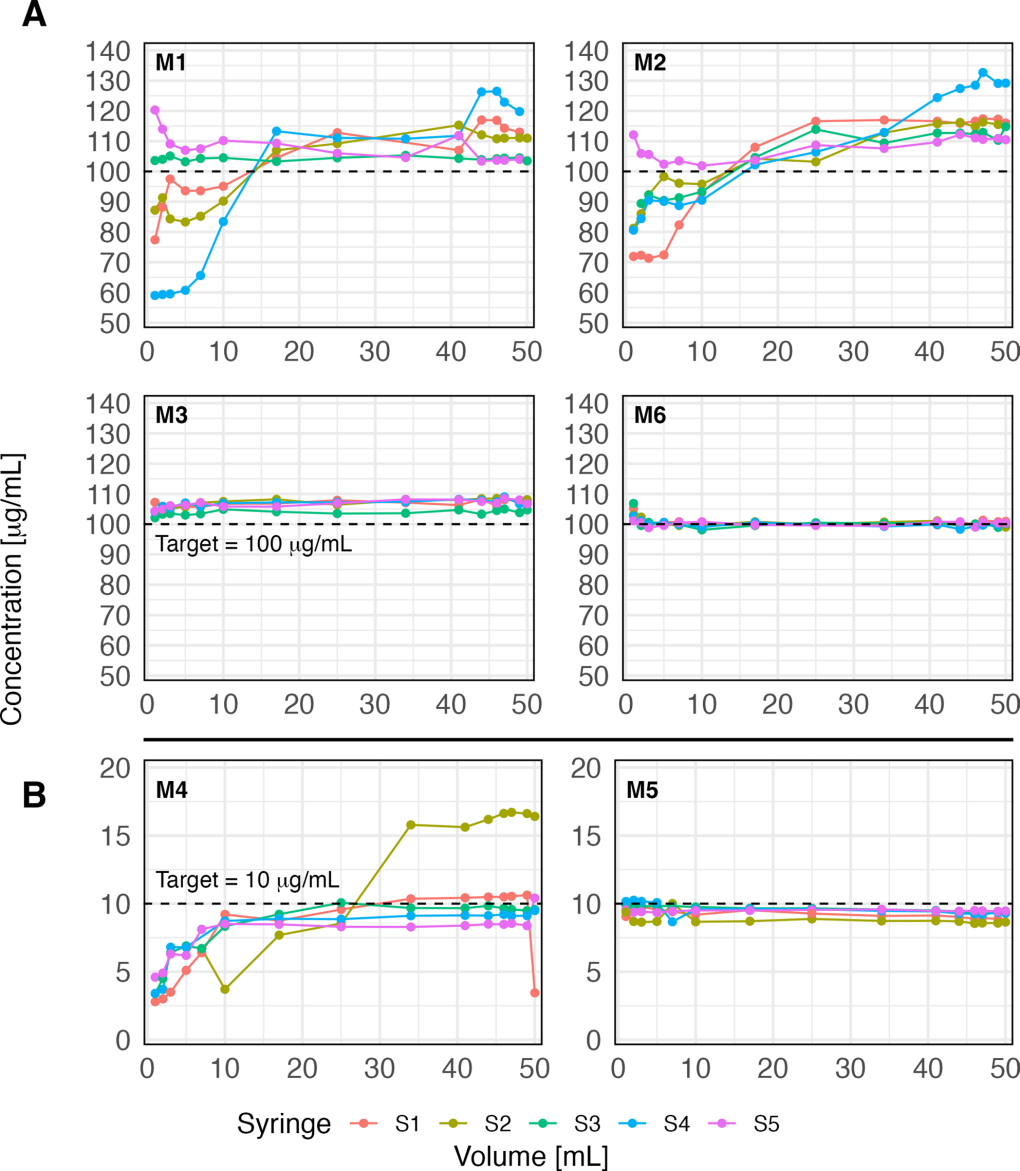
Noradrenaline concentration profiles in 50 mL syringes from syringe pump experiments. Each preparation method (M1-M6) was replicated five times (S1-S5). **A**: methods targeting 100 µg/mL (M1-M3, M6); **B**: methods targeting 10 µg/mL (M4-M5). Mixing technique: No-Mix (M1, M4), Flip-Only (M2), Bubble-Flip (M3, M5), Pre-Mix (M6)

NA concentration profiles for all preparation methods (M1-M6) and replicate experiments (S1-S5) are shown in Fig. 3. At the method level, variability (expressed as CV) ranged from 0.90 % to 30.7 %. No-Mix method M4 showed by far the highest variability (30.7 %), followed by the Flip-Only method M2 (12.1 %) and the second No-Mix method M1 (9.2 %). In contrast, the Bubble-Flip method M3 (0.90 %) and the Pre-Mix formulation M6 (1.0 %) showed the lowest variability, with the Bubble-Flip method M5 (2.0 %, low concentration) only slightly higher.

**Fig. 4:**
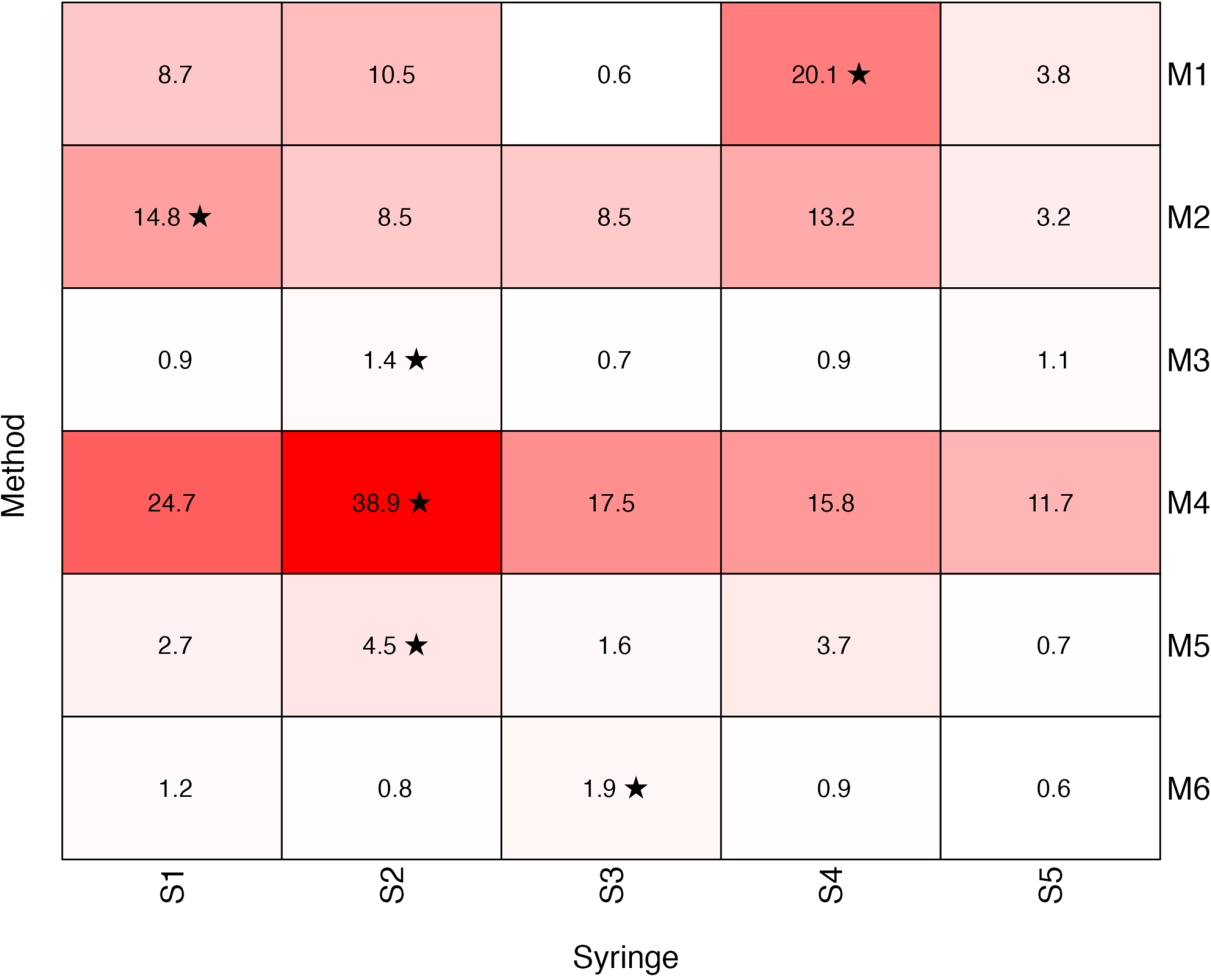
Within-syringe variability of noradrenaline concentrations from syringe pump experiments shown as heat map of coefficients of variation as percentage. Results are displayed for all preparation methods (M1-M6) and replications (S1-S5). Mixing technique: No-Mix (M1, M4), Flip-Only (M2), Bubble-Flip (M3, M5), Pre-Mix (M6). ★: highest variability per method

**Fig. 5:**
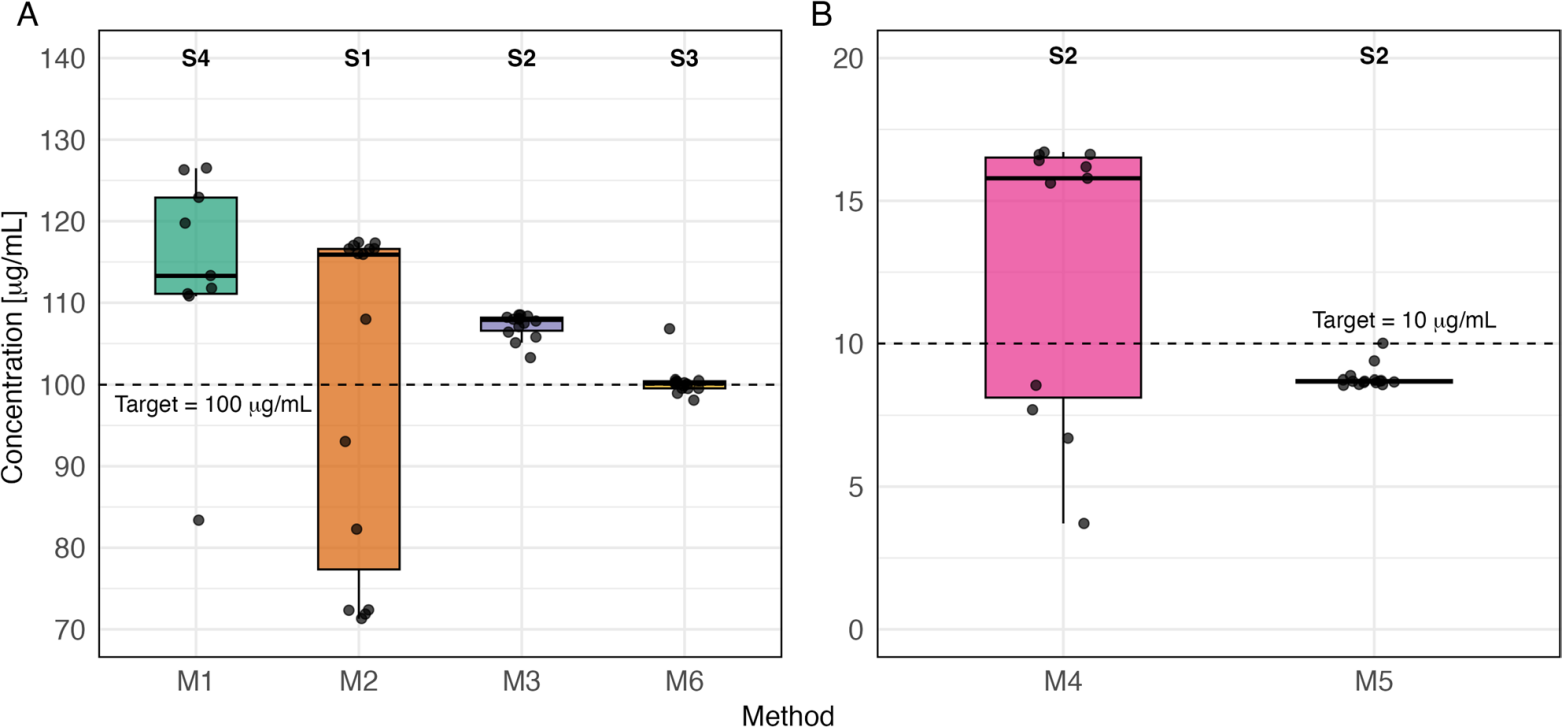
Noradrenaline concentrations in the single most variable syringe pump experiment per preparation method (M1-M6), shown as boxplots. **A**: methods targeting 100 µg/mL (M1-M3, M6); **B**: methods targeting 10 µg/mL (M4-M5). Mixing technique: No-Mix (M1, M4), Flip-Only (M2), Bubble-Flip (M3, M5), Pre-Mix (M6)

Within-syringe variability (expressed as CV) across all 30 syringe pump experiments ranged from 0.61 % to 38.9 % (Fig. 4). The least homogeneous syringes were M4 (No-Mix, S2, 38.9 %), M1 (No-Mix, S4, 20.1 %), and M2 (Flip-Only, S1, 14.8 %), whereas much lower CVs were observed in M3 (Bubble-Flip, S2, 1.4 %), M6 (Pre-Mix, S3, 1.9 %), and M5 (Bubble-Flip, S2, 4.5 %) (boxplots in Fig. 5).

The proportion of individual measurements deviating by more than $\:\pm\:$15 % from the target concentration ranged from 0.0 % to 52.9 %, depending on the preparation method. The highest proportions were found in M4 (No-Mix, 52.9 %), M2 (Flip-Only, 35.6 %), and M1 (No-Mix, 24.3 %), whereas no deviations beyond this threshold were detected in M3 (Bubble-Flip), M5 (Bubble-Flip), or M6 (Pre-Mix) (Table 2).

**Table 2:** Large deviations from target noradrenaline concentration in syringe pump experiments

Preparation method	Total samples	Samples exceeding tolerance	Proportion
	$\:{N}_{\mathrm{total}}$	$\:{N}_{\mathrm{>}15{\%}}$	[%]
M1 (No-Mix)	70	17	24.3
M2 (Flip-Only)	73	26	35.6
M3 (Bubble-Flip)	74	0	0.0
M4 (No-Mix)	70	37	52.9
M5 (Bubble-Flip)	73	0	0.0
M6 (Pre-Mix)	75	0	0.0

Frequency of deviations greater than $\:\pm\:$15 % from target noradrenaline concentration across 30 syringe pump experiments. Each row shows the preparation method (M1-M6), the number of samples analysed $\:{N}_{\mathrm{total}}$, the count of samples exceeding the tolerance limit$\:{N}_{\mathrm{>}15{\%}}$, and the corresponding proportion

### Flash-freeze experiments

**Fig. 6:**
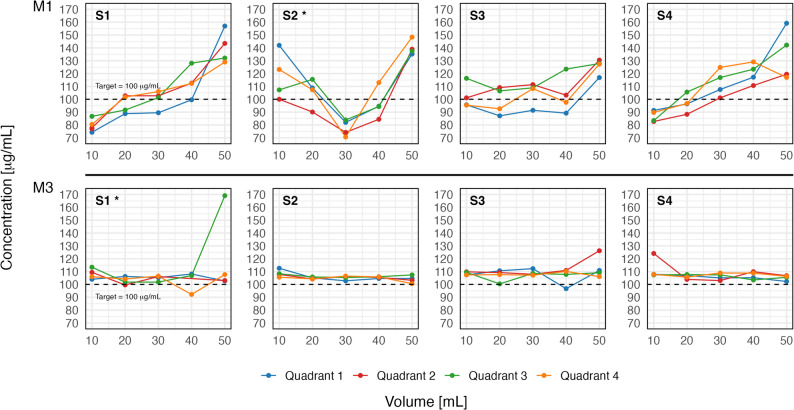
Noradrenaline concentration profiles in 50 mL syringes subjected to flash-freezing. Profiles are shown for solution preparation methods M1 (No-Mix) at the top row and M3 (Bubble-Flip) at the bottom. Each profile represents measurements across quadrants (1–4) within the respective 10 mL syringe section (S1-S4). *: experiment with highest variability per method

**Fig. 7:**
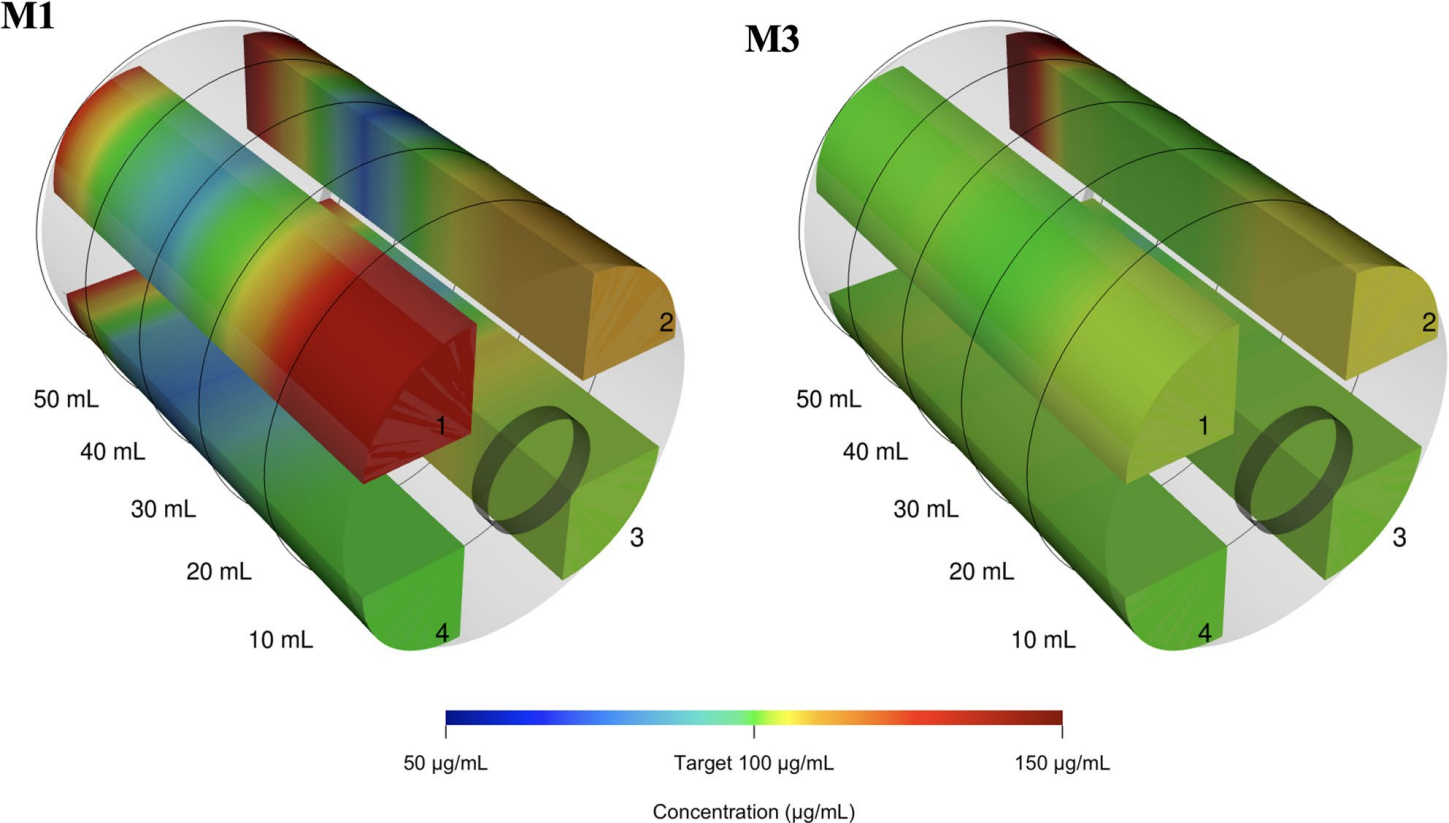
Three-dimensional reconstructions of noradrenaline concentrations in the two most inhomogeneous flash-freeze experiments. Quadrants (1–4) are displayed as visually separated parts for illustration purposes. M1: preparation method M1 (No-Mix), syringe S2; M3: preparation method M3 (Bubble-Flip), syringe S1

NA concentration profiles for all preparation methods M1 and M3, including all quadrants and replicates (S1-S4), are shown in Fig. 6. Within-syringe CVs for both methods are reported in Table 3. The linear regression model showed no evidence of an association of within-syringe CV with storage time before quantification for M1 ($\:p\mathrm{=}0.52$) and M3 ($\:p\mathrm{=}0.34$), and the slopes did not differ between methods (interaction $\:p\mathrm{=}0.81$). Three dimensional reconstructions of the replicates with the highest CV in each method (highlighted by * in Fig. 6) are illustrated in Fig. 7.

**Table 3:** Results of flash-freezing experiments for in vitro noradrenaline preparations

Method	Syringe	Storage time [min]	Coefficient of variation [%]
M1	S1	0	21.7
M1	S2	30	22.1
M1	S3	60	12.6
M1	S4	120	18.2
M3	S1	0	14.2
M3	S2	30	2.3
M3	S3	60	5.0
M3	S4	120	4.2

Variability of noradrenaline concentrations as coefficients of variation for two preparation methods: M1 (No-Mix) and M3 (Bubble-Flip). Four syringes (S1-S4) per method were analysed after storage at room temperature for 0–120 min before quantification

## Discussion

The preparation of drug solutions is a routine yet critical task in anaesthesiology and intensive care, often performed under time constraints and without standardised mixing protocols. However, inadequate mixing can lead to concentration inhomogeneities that compromise dosing accuracy, therapeutic efficacy, and also our perception of the patients’ clinical condition. Despite these risks, the adequacy of mixing practices is rarely questioned, let alone scrutinised in depth: for the clinician, prepared solutions are typically beyond doubt and assumed to be homogeneous. In this study, we systematically compared six preparation methods for noradrenaline solutions in 50 mL syringes, including two clinically relevant concentrations (0.1 mg/mL and 0.01 mg/mL), three mixing techniques (*No-Mix*, *Flip-Only*, *Bubble-Flip*), and one commercial pre-mixed solution.

The mixing techniques *No-Mix*, defined as the absence of any deliberate step to promote mixing of the two components, and *Flip-Only*, which adds a single end-over-end inversion (360°) without an air bubble, both resulted in pronounced concentration inhomogeneities (Figs. 3, 4 and 5). The highest overall method-level CVs for NA concentrations were 30.7 % (M4) and 12.2 % (M2), even though these represent conservative estimates derived from values averaged across replicates at each sampling point. Also, a substantial proportion of samples deviated by more than $\:\pm\:$15 % from the intended target concentration for both mixing techniques (Table 2). Accordingly, both mixing techniques must be regarded as unsuitable for clinical use. In contrast, the *Bubble-Flip* mixing technique - i.e. aspirating an additional 5 mL of air followed by a single end-over-end inversion - consistently yielded homogeneous solutions, with CVs ≤2 % and no deviations greater than $\:\pm\:$15 % from target concentrations. The commercial pre-mixed solution (Sinora^®^ noradrenaline 0.1 mg/mL) performed equally well.

Variability in drug concentration within syringes directly translates into variability of drug delivery when administered via infusion pumps. For slow-acting drugs with wide therapeutic margins (e.g. antibiotics), such variability may be clinically irrelevant. For short-acting drugs with steep dose-response curves such as vasopressors, however, even moderate inhomogeneities can provoke abrupt pharmacodynamic effects, necessitating repeated dose adjustments, and potentially end-organ complications due to hypoperfusion or hypertension. The European Pharmacopoeia allows a content variation of typically $\:\pm\:$15 %, providing a pragmatic benchmark for interpreting our results [10]. While such deviations in overall syringe content are often clinically negligible, particularly because vasopressors are titrated to effect and infusion rates are continuously adjusted, inhomogeneity within the syringe presents a fundamentally different problem. Even when the mean concentration is acceptable, transient spikes or troughs caused by poor mixing can lead to abrupt changes in drug delivery. Importantly, such concentration inhomogeneities often remain hidden in daily practice. Apparent fluctuations in haemodynamic response are usually attributed to the patient’s condition, while the possibility of inadequate mixing is rarely considered. Misinterpretation of these effects may trigger cascades of diagnostic procedures and unnecessary interventions (e.g. administration of intravenous fluids or additional vasopressors), ultimately compromising patient safety.

Given the pivotal role of noradrenaline dosing as inclusion or stratification criterion in septic shock trials - whether directly or indirectly through instruments such as the SOFA-score [11–13] - and its influence on therapeutic recommendations (e.g. initiation of vasopressin at noradrenaline doses in the range of 0.25–0.5 µg/kg/min [14], inconsistencies in dose reporting add another layer of uncertainty about actually delivered doses. Furthermore, the lack of harmonisation regarding noradrenaline salt formulations has been highlighted in recent years [15]. Although the absolute effect of imperfect syringe mixing in our experiments was smaller than the systematic two-fold error that occurs when norepinephrine tartrate and base are confused, its clinical relevance may be even greater owing to its variability and the somewhat unpredictable consequences it introduces.

Our dense sampling strategy provided detailed insight into where marked inhomogeneities arise most frequently. Variability was most pronounced at the ends of syringes (Fig. 3), likely reflecting layering due to inadequate mixing [16]. This observation has direct clinical implications: syringe exchanges are common points of haemodynamic instability in patients receiving high-dose vasopressors. Such changes are often dismissed as transient effects of infusion interruption; however, our data indicate that the initial millilitres of a newly connected syringe may contain markedly lower drug concentrations, followed by steep overshoots above target levels. This sequence can amplify apparent haemodynamic instability rather than merely reflect patient physiology. Three-dimensional profiling further demonstrated that inhomogeneities are not limited to the longitudinal axis but also occur radially across the syringe cross-section, underscoring the risk of unpredictable fluctuations during continuous infusions. Mixing quality was also influenced by specific preparation conditions: lower drug-to-solvent ratios increased the likelihood of inhomogeneity, particularly when small volumes of concentrated drug were added to large diluent volumes without subsequent mixing (compare M4 and M5 against M1 and M3, Fig. 4). Furthermore, storage without agitation showed no detectable effect on homogenization; there was no evidence that solutions self-homogenize over time. As expected, solutions remained stratified unless actively mixed, emphasising that proper mixing at preparation is indispensable. It should also be noted that several other dilution techniques in common clinical use, including variants not evaluated in our experiments, may achieve similarly good mixing performance when applied correctly.

Medication errors are common, as demonstrated by multiple systematic reviews [17–20]. However, both research and system-level measures can reduce risk [21–27]. Errors related to medication also carry a substantial economic burden [28, 29]. Vulnerabilities arise throughout the medication process from factors such as interruptions, workload, insufficient training, and a lack of standardised procedures [1, 30]. Safety has improved through strategies like standardisation, checklists, and safeguards for high-alert medications [31, 32]. While prescribing and administration errors are well studied, the preparation step is an equally critical source of error [3, 33, 34]. Unlike more visible errors, preparation errors often remain undetected and their contribution to harm overlooked - an issue our study directly addresses.

Our findings extend prior work on inadequate mixing in several ways [7–9, 16, 35–37]. First, we applied high-resolution sampling. Second, we demonstrated concentration gradients in a three-dimensional reconstruction. Third, we systematically evaluated the role of an air bubble with a single inversion (the *Bubble-Flip* method) for an anaesthesiology-relevant critical drug. Finally, by comparing worst-case syringes across preparation methods, we emphasised what is clinically most important in high-acuity settings: consistency. A method that “usually works” cannot be considered safe if it occasionally fails catastrophically. Based on our study, we are sufficiently convinced of the robustness of the *Bubble-Flip* method. Where acquisition costs are not a limiting factor, the industrially pre-mixed solution is certainly the most robust alternative.

Our study has several limitations. First, although the in vitro design allowed us to work under controlled conditions, it only partly reflects bedside practice and does not include patient-oriented clinical endpoints. In routine anaesthesiology and intensive care, manual syringe preparation by multiple operators introduces additional variability that our experiments did not capture. Instead, we focused on interpreting worst-case scenarios, which we consider highly relevant from a patient safety perspective. Second, it remains uncertain whether drugs with substantially different physicochemical properties would behave in the same way. We investigated only noradrenaline in normal saline, albeit at two clinically relevant concentrations and with several preparation techniques. Nevertheless, noradrenaline - being the most widely used short-acting vasopressor worldwide - should represent a model drug of practical significance. Third, our three-dimensional reconstructions were based primarily on longitudinal gradients, while the transverse axis was represented by homogeneous representation within quadrants of each cross-section. This simplification was deemed sufficient for illustration. Fourth, we investigated only a subset of the dilution and preparation methods used in clinical practice; other commonly employed techniques may provide comparable homogeneity and were not formally assessed in this study. Given these limitations, further investigations under real-world conditions could be of value, particularly if they assess both preparation quality and pharmacodynamic consequences in patients.

Overall, our findings show that manual preparation of diluted noradrenaline solutions can yield unacceptable concentration inhomogeneities within individual syringes, posing a latent risk to patient safety. This risk can be reliably mitigated either by using pre-mixed products or by inverting the syringe end-over-end in the presence of a 5 mL air bubble.

## Conclusion

Drug preparation in anaesthesiology and intensive care must be reliable, not just “usually adequate”. The mixing technique has a decisive impact on the homogeneity of noradrenaline solutions in 50 mL syringes. Our study demonstrates that even simple, low-effort interventions - such as the *Bubble-Flip* method - can reliably achieve clinically acceptable concentrations. These findings highlight the importance of recognising preparation as a critical step in medication safety and provide a practical basis for institutions to decide between standardised in-house protocols and industrially pre-mixed solutions.

## Data Availability

The datasets used and analysed during the current study are available from the corresponding author on reasonable request.

## Abbreviations

NA: Noradrenaline
CV: Coefficient of Variation
NS: Normal Saline
HPLC: High Performance Liquid Chromatography
SOFA: Score Sequential Organ Failure Assessment score
